# Thyroid diseases in children and adults with celiac disease: A cross-sectional study

**DOI:** 10.22088/cjim.15.2.307

**Published:** 2024

**Authors:** Ramin Niknam, Heydar Baseri, Laleh Mahmoudi, Mohammad Reza Fattahi, Ebrahim Fallahzadeh Abarghooei, Ali Zamani

**Affiliations:** 1 Gastroenterohepatology Research Center, Shiraz University of Medical Sciences, Shiraz, Iran; 2 Department of Clinical Pharmacy, School of Pharmacy, Shiraz University of Medical Sciences, Shiraz, Iran; 3 Endocrine and Metabolism Research Center, Shiraz University of Medical Sciences. Shiraz, Iran

**Keywords:** Thyroid disease, Celiac disease, Children, Adults, Serology, Histology.

## Abstract

**Background:**

There are few reports evaluating different factors, including the severity of duodenal histopathological findings and serological levels of celiac disease (CD), in increasing the probability of thyroid diseases (TD) in adults and children with CD, so, we designed this research.

**Methods:**

CD was defined as Marsh type 2 or higher in duodenal histopathology and serological levels of anti-transglutaminase antibodies (anti-tTG) equal to or greater than 18 IU/ml. To assess the likelihood of TD in CD patients, logistic regression analysis was employed.

**Results:**

538 patients were included in this study. Of these, 354 (65.8%) were females and 184 (34.2%) were males. 370 (68.8%) patients were children. Overall, 57 (10.6%) patients had TD, of which 49 (9.1%) had hypothyroidism and 8 (1.5%) had hyperthyroidism. Adults had a significantly higher probability of developing TD than children (OR 1.9; 95% CI 1.1-3.4; P = 0.03). The odds of developing TD were also significantly higher in patients with family marriage in parents (OR 2.3; 95% CI 1.1-4.7; P = 0.03). Other variables such as gastrointestinal symptoms, anti-tTG levels, and severity of Marsh classification did not exhibit a substantial rise in the likelihood of TD development.

**Conclusion:**

The study findings indicated that the likelihood of developing TD in CD patients can be linked to advancing age and having family marriage in parents, while there was no significant association observed with anti-tTG levels, severity of histological damage, and gastrointestinal symptoms.

**C**eliac disease (CD) is a common autoimmune enteropathy caused by an abnormal immune response to the gliadin protein (1-3). CD affects around 1% of the general population (4) and is associated with various immune-mediated disorders, such as thyroid diseases (TD) (5, 6). TD is a common disorder, but comparing prevalence and incidence between countries is challenging (7, 8). Studies show a higher incidence of TD in individuals with CD compared to the general population (9, 10). The pathogenesis of co-existing TD and CD is unknown, but some hypotheses have been proposed (10). 

Screening for TD in CD patients is recommended (9). However, screening is difficult and costly due to the high prevalence of both CD and TD in the general population (7, 8, 11). Identifying risk factors for the coexistence of these two diseases is crucial for targeted screening (12). This study aimed to investigate the different types of TD in children and adults with CD and also to evaluate various independent variables in increasing the chances of TD in CD. 

## Methods

**Ethical approval/statement:** This research was approved by the Ethics Committee of Shiraz University of Medical Sciences (ID number: 21899; IR.SUMS.MED.REC.1399.633) based on Helsinki Declaration of Ethics for Medical Research, after review by the institutional board.

All CD patients were required to provide written consent before their medical records could be reviewed.

**Population:** The prevalence of TD and the effect of different factors on raising the probability of TD in CD patients were examined in this cross-sectional study from December 2015 to October 2022. All participants referred to the celiac clinic whose information was recorded in the Fars Celiac Registry were evaluated according to the inclusion and exclusion criteria of the study (Approval ID: IR.SUMS.REC.1399.525). A checklist was used to gather information on age, sex, personal and family medical history, histological reports, IgA anti-transglutaminase antibodies (anti-tTG) levels, thyroid-stimulating hormone (TSH) levels, and free thyroxine (free T4) levels. Individuals who were older than 19 years were categorized as adults, while those who were 19 years old or younger were categorized as children. At the end, we compared various variables, including demographic, clinical, histological, and serological findings, between patients with and without TD.

**Laboratory tests:** Anti-tTG levels were measured by ELISA method using Aeskulisa kit (Germany). Specimens collected from the duodenum were subjected to hematoxylin/eosin staining and evaluated based on the Oberhuber-modified Marsh classification for histological interpretation (13). The level of TSH was measured by chemiluminescent microparticle immunoassay using TSH CLIA Microparticles (intra-assay coefficient of variation of 2.5% and an inter-assay of 5.3%; reference ranges: 0.35-5.50 mIU/mL; Belgium). The level of free T4 was measured by radioimmunoassay using the Elisa Kit.Ideal (intra-assay coefficient of variation of 4.7% and an inter-assay coefficient of variation of 5.9%; reference ranges: 0.8-2 ng/dL; Iran).

**Definitions of celiac and thyroid diseases:** The criteria for diagnosing CD involved an anti-tTG level of ≥18 IU/mL and the presence of Marsh type 2 or higher on histological examination (4, 13-15). All participants were on a gluten-containing diet, and the interval between serological testing and duodenal biopsy was less than one month. The diagnosis of CD was confirmed by a gastroenterologist. CD patients were then evaluated for TD by an endocrinologist, which was classified into three groups: hypothyroidism (TSH > 5.50 mIU/mL), euthyroid (TSH between 0.35–5.50 mIU/mL), and hyperthyroidism (TSH <0.35 mIU/mL).

**Inclusion and exclusion criteria:** All children and adults with CD meeting the aforementioned definition were considered potential participants in the study. However, patients who did not cooperate with the study, those on a gluten-free diet, those with diseases that could affect thyroid function tests (such as tumors, trauma, infiltrative diseases, metabolic or systemic disorders affecting pituitary or hypothalamic function), those taking medications that could affect thyroid function tests (such as phenobarbital, valproate, phenytoin, carbamazepine, rifampin, furosemide, nonsteroidal anti-inflammatory drugs, and protease inhibitors), pregnant women, those with IgA deficiency, those with Marsh type 0 or 1, or those with other causes of villi atrophy were excluded from the study.

**Statistical analysis:** Data were collected and analyzed in SPSS software (USA) Version 25. Continuous variables were expressed as mean ± standard deviation (SD) and independent t-test was used for analysis. Categorical variables were presented as frequency/percentage and chi-square test was used to compare between groups. Non-parametric tests were used when the Kolmogorov-Smirnov test indicated significance for variables. We used logistic regression analysis to estimate the odds ratios (ORs) of different independent factors in the development of TD. A p- value < 0.05 was considered statistically significant.

## Results

The data of 538 patients were analyzed. The mean (±SD) age of the patients was 18.8 (±14.5) years. The age range was 3 to 70 years. Of the total, 354 (65.8%) were females, and 184 (34.2%) were males. The participants were further divided into child (n=370, 68.8%) and adult (n=168, 31.2%) groups. The mean (±SD) anti-tTG level was 210.9 (±204.6) IU/mL. The mean (±SD) levels of free T4 and TSH were 1.3 (±2.0) ng/dL and 3.4 (±4.1) mIU/mL, respectively. Table 1 summarizes the demographic, clinical, and paraclinical characteristics of the participants (table 1).

Out of 538 patients, 57 (10.6%) had TD, of which 49 (9.1%) had hypothyroidism and 8 (1.5%) had hyperthyroidism. The comparison of demographic, clinical, and paraclinical characteristics of participants with and without TD is presented in table 2. The mean age of TD patients (23.3±16.1 years) was significantly higher than in euthyroid participants (18.2±14.2 years). Frequency of TD in women (11.6%) was relatively higher than in men (8.7%), but this difference was not statistically significant. Frequency of family marriage in parents was significantly higher in patients with TD (21.1%) than euthyroid participants (8.9%) (table 2). Impact of different independent variables on TD was examined through logistic regression analysis (table 3). The analysis revealed that adults had a significantly higher probability of developing TD than children group (OR 1.9; 95% CI 1.1-3.4; P = 0.03). CD patients with a familial marriage in parents also had a significantly higher probability of developing TD than participants without familial marriage in parents (OR 2.3; 95% CI 1.1-4.7; P = 0.03). Other variables did not increase the chance of TD, including the severity of marsh classification and levels of anti-tTG (table 3). 

**Table 1:** Characteristics of celiac disease participants (n=538)

**Varibles**	
Age (yrs.); Mean ± SD	18.76**±**14.47
Gender; N (%)	
Male Female	184 (34.2%) 354 (65.8 %)
Ethnicity; N (%)	
Fars Lure Turk Others	419 (77.9%) 74 (13.8%) 30 (5.6%) 15 (2.8%)
Anti-transglutaminase antibodies (IU/ml) ; Mean ± SD	210.85**±** 204.57
Thyroid-Stimulating Hormone (mIU/mL) ; Mean ± SD	3.36±4.13
Free thyroxine (T4); (ng/dl); Mean ± SD	1.33±2.02
Gastrointestinal symptoms; N (%)	344 (63.9%)
Thyroid disease; N (%)	
Normal Hypothyroidism Hyperthyroidism	481 (89.4%) 49 (9.1%) 8 (1.5%)
Marsh classification ^1^; N (%)	
Marsh 2 Marsh 3a Marsh 3b Marsh 3c	25 (4.6 %) 169 (31.4%) 220 (40.9%) 124 (23.0 %)
Celiac disease in the family; N (%)	44 (8.2%)
Family marriage in the parents; N (%)	55 (10.2%)

Notes: 1 Histopathological findings of duodenum were classified based on Oberhuber-modified Marsh classification

**Table 2:** Demographic, clinical, and paraclinical findings in celiac disease patients with and without thyroid disease (n=538)

**Variables**	**Without thyroid disease; (n=** **481 )**	**With thyroid disease; (n=** **57)**	P value*
**Age **(yrs.); Mean ± SD^1^	18.23±14.19	23.25±16.10	0.028
**Age ** ^2^; n (%)			
Children Adults	340 (91.9%) 141 (83.9%)	30 (8.1%) 27 (16.1%)	0.005
**Gender ** ^2^ **;** n (%)			
Male Female	168 (91.3%) 313 (88.4%)	16 (8.7%) 41 (11.6%)	0.302
Ethnicity^2^			
Fars Lure Turk Others	374 (77.8%) 67 (13.9%) 27 (5.6%) 13 (2.7%)	45 (78.9%) 7 (12.3%) 3 (5.3%) 2 (3.5%)	0.971
**Gastrointestinal symptoms ** ^2^	304 (63.2%)	40 (70.2%)	0.300
**Celiac disease in the family** ^2 ^	40 (8.3%)	4 (7.0%)	0.735
**Family marriage in parents** ^2 ^;n (%)	43 (8.9%)	12 (21.1%)	0.004
**Marsh classification** ^2,4^			0.187
Marsh 2	24 (5.0%)	1 (1.8%)	
Marsh 3a	152 (31.6%)	17 (29.8%)	
Marsh 3b	190 (39.5%)	30 (52.6%)	
Marsh 3c	115 (23.9%)	9 (15.8%)	
**Anti-transglutaminase antibodies ** ^5^ (IU/mL) ; Mean ± SD^3^	211.24±207.25	207.51±182.06	0.672

Notes: ^1 ^Independent sample t-test; ^2 ^Chi-square test; ^3 ^Mann-Whitney Test; ^4 ^Histopathological findings of duodenum were classified based on Oberhuber-modified Marsh classification.

**Table 3:** Impact of different variables on thyroid disease using logistic regression analysis (n=538) for estimating odds ratio (OR)

**Variable**	**Univariate analysis**		**Multivariate analysis**	
	**OR (95% confidence interval)**	**P value**	**OR ** **(95% confidence interval) **	**P-value**
**Age **				
Adults Children	2.170 (1.245-3.783) 1.0	0.006	1.923 (1.085-3.409) 1.0	0.025
**Gender**				
Male Female	1.375(0.749-2.525) 1.0	0.304	0.733 (0.396-1.357) 1.0	0.323
**Family marriage in parents**				
Yes No	0.368 (0.181-0.749) 1.0	0.006	2.249 (1.082-4.677) 1.0	0.030
**Gastrointestinal symptoms**				
Yes No	0.730 (0.402-1.326) 1.0	0.301	1.214 (0.652-2.259) 1.0	0.541
**Histological findings** ^1^				
Non-atrophic Atrophic	2.941 (0.390-22.159) 1.0	0.295	0.326 (0.042-2.501) 1.0	0.281

Notes: ^1 ^Severity histopathology of the duodenal biopsies was classified into 2 subgroups of non-atrophic and atrophic (Marsh 3a, 3b, 3c) based on Oberhuber-modified Marsh classification.

## Discussion

This study in CD patients showed that the probability of TD was significantly higher in adults than in children. Additionally, familial marriage in parents increased the probability of developing TD in CD patients. The severity of duodenal villous atrophy and the level anti-tTG did not affect the chance of developing TD.

CD is a common autoimmune disorder that damages the small intestine and is associated with various non-gastrointestinal disorders (15, 16). The prevalence of CD is around 1%, with a higher incidence in women than in men (4). Gluten consumption and genetic susceptibility are the main risk factors for developing CD symptoms (17-20). TD is a global health issue that significantly impacts health. 

Prevalence of hypothyroidism in general population of United States and Europe ranges from 0.3% to 3.7% and from 0.2% to 5.3%, respectively. On the other hand, in iodine-replete communities, the prevalence of hypothyroidism is 1 to 2%, which is more common in elderly women (7, 8). It is difficult to compare the prevalence of TD in different countries due to differences in population selection, iodine nutrition, diagnostic thresholds, and assay sensitivities. Both adults and children with CD have a strong association with TD (9-11). The underlying mechanisms for this relationship are unknown, but several hypotheses have been proposed. In CD patients who are consuming a gluten-containing diet, a loss of intestinal barrier integrity with changes in the systemic immune response may occur. CD and TD may also share one or more genes, which could be another explanation for this association (10).

In this study, the likelihood of developing TD in adults was almost two times higher than in children. While the majority of participants in this study were women, there was no notable gender difference concerning the development of TD. In a study from Iran, TD was significantly more common in women than in men (15% vs. 10% in patients, and 4.3% vs. 1.2% in controls). Furthermore, the frequency of occurrence was greater among younger individuals as compared to adults (16% vs. 12.5%) (12) , which is inconsistent with our results. In some other studies, the prevalence of TD in CD was higher in children than in adults (12, 21). The different results between our study and previous research may be related to differences in study design and methods, including inclusion and exclusion criteria, sample size, and study location. 

To the best of our knowledge, there is no study to determine the association between anti-tTG level and histological severity for the development of TD in CD patients. In our research, Marsh type 3b was the most common histopathological finding, but the severity of CD was not related to the probability of TD occurrence. Similarly, anti-tTG levels was not related to the probability of developing TD. Therefore, histological severity and anti-tTG levels may not be reliable indicators for selecting CD patients for TD screening until further research is conducted.

Interestingly, this study found that the presence of family marriage in parents increased the probability of developing TD by almost 2.3 times. This results could potentially be linked to genetic factors, and additional research is suggested to provide clarity on this matter. Although gastrointestinal symptoms were more prevalent in the TD patients than the non-TD participants, no significant difference was observed between them. Therefore, gastrointestinal symptoms are not currently an indicator for the evaluation of TD in CD until further investigation. One strength of this study was the comparison of different independent variables in development of TD in both age of groups of children and adults with CD. Other strengths included the acceptable sample size and the presence of detailed demographic, serological, and histological characteristics for all participants. Limitations of this study included the absence of a control group and the lack of thyroid peroxidase antibodies measurement.

To sum it up, this study showed that increasing age and familial marriage in parents could increase the probability of TD in CD patients. In contrast, anti-tTG levels, histological severity, and gastrointestinal symptoms did not increase the probability of TD development. Additional investigation is warranted to verify these results.
